# Latent dependency classes according to the need for help: a population-based analysis for the older population

**DOI:** 10.1186/s12877-022-03307-w

**Published:** 2022-07-26

**Authors:** Alejandra Marroig, Guillermo Sánchez-Laguardia, Maira Colacce, Julia Córdoba, Graciela Muniz-Terrera

**Affiliations:** 1 Instituto de Estadística, Universidad de la República, Gonzalo Ramírez 1926, Montevideo, 11200 Uruguay; 2 Instituto de Economía, Universidad de la República, Montevideo, Uruguay; 3 Facultad de Psicología, Universidad de la República, Montevideo, Uruguay; 4 Department of Social Medicine, Ohio University, Athens, USA; 5 University of Edinburgh, Edinburgh, UK; 6 BrainLat, Universidad Adolfo Ibañez, Santiago, Chile

**Keywords:** Care dependency, Activities of daily living, Latent class approach, Older adults, Developing countries

## Abstract

**Background:**

Older adults living in the community may have daily needs for help to perform different types of activities. In developing countries, older adults face the additional challenge of lacking sufficient economic means to face their increasing needs with ageing, and health and social policies may be under pressure. The aim of this study was to assess dependency in the older population from a developing country using a latent class approach to identify heterogeneity in the type of activities in which dependent older adults require help.

**Methods:**

In this cross-sectional evaluation of dependency, we considered individuals aged 60 years and older from a nationally representative study (*N* = 5138) in Uruguay. We fitted latent class regressions to analyse dependency, measured by the need for help to perform Activities of Daily Living, adjusted by sociodemographic characteristics.

**Results:**

Four latent classes were identified, 86.4% of the individuals were identified as non-dependent, 7.4% with help requirements to perform instrumental activities while individuals in the other two classes need help to perform all types of activities with different degrees (4.3 and 1.9%). Less educated women are more likely to be in the group with needs in instrumental activities.

**Conclusions:**

The heterogeneous patterns of dependency have to be addressed with different services that meet the specific needs of dependent older adults.

**Supplementary Information:**

The online version contains supplementary material available at 10.1186/s12877-022-03307-w.

## Background

Health and social care systems are often under pressure due to the higher number of adults who reach old age and experience poorer health, older disability, and dependency [1, 2]. Developing countries face the additional challenge of insufficient economic resources to provide for these increasing demands in services, which may hamper the opportunity to ensure an adequate quality of life in old age [3]. Moreover, the increases in the older segments of the population could place additional pressure on the demand for social care for older adults living at home, which may be the preferred living option for a significant portion of people as they age [4, 5]. Concomitantly, the difficulties and need for assistance of the older population are often related to differences in their sociodemographic characteristics [1, 6–10]. Therefore, a better understanding of how these disparities in individual characteristics may result in differences in the need for support may provide evidence for improving policies aimed at older adults.

The health of older adults is characterized by the co-occurrence of morbidities, disability, and frailty, which are also associated with a more intensive use of social care support and more dependency [11, 12]. Dependency, a multidimensional construct, is defined as the need for human help or care to perform Activities of Daily Living (ADL) [13] and is highly prevalent in the older population. The operationalisation of dependency is not consistent in the literature, as different types of activities are used to define this concept. For instance, some use Basic Activities of Daily Living (BADL) [14] when an individual is dependent, whereas others use Instrumental Activities of Daily Living (IADL) [15], and Advanced Activities of Daily Living (AADL) [16]. These differences suggest that an improved understanding of the need for help according to the different types of activities in which individuals need it, may help to design better programs oriented to the specific needs of the older adult population who are dependent.

Uruguay is a Latin American country that has experienced significant progress in the demographic transition resulting in a mature age structure, with 19% of the population aged 60 years or older in 2015 [17].

Regarding policies aimed at the dependent population, Uruguay stands out among developing countries for having a regulatory framework for these policies, named *Sistema Nacional Integrado de Cuidados* (SNIC, The National Integrated Care System) [18]. The SNIC in Uruguay regulates the care services provided to the population of dependent individuals, determines rights and obligations of beneficiaries, and manages and executes services and programs for this population [19]. Although the SNIC defines the dependent population as the group of individuals who needs help from others to perform ADL, regardless of age, our work focuses on dependency of older adults.

The SNIC provides Personal Assistants, Tele-assistance and Day Care Centres [20]. The Tele-assistance and Day Care Centres are services provided to individuals with mild to moderate dependency and the Personal Assistants benefit is directed to severely dependent individuals living at home. None of these benefits consider the specific activities the person needs support with, only the severity of the dependency. For instance, the Personal Assistants program subsidizes 20 hours per week of a caregiver’s time, regardless of the type of daily activities in which individuals may need help [20]. SNIC services are allocated according to an instrument called *Baremo de dependencia* (BAREMO hereinafter) developed in 2013 [21]. BAREMO measures the need for help from another person, based on the reason for needing help (performance problem), the type of help needed, the domain in which help is needed (physical, mental, or both reasons), and the frequency in which the help is needed. The instrument is based on the assessment of 13 Activities of Daily Living, including BADL, IADL, and AADL, each one disaggregated into specific tasks. As a result of the assessment of these activities, a score is obtained on a continuous scale. BAREMO does not require a prior assessment of the disability or certified diagnoses, rather, it is based on the daily assistance needs and on the interviewees’ narrative about the ability to perform the activity.

Additionally, the SNIC has developed cut-off points of the BAREMO scale for the allocation of potential beneficiaries to different programs according to the severity of their dependency (non-dependent, mild-dependent, moderate-dependent, and severe-dependent). Further, the instrument has been adapted to utilize the information available in a population-based survey conducted in Uruguay [21], and this adaptation includes almost all the activities assessed in the original scale, without the disaggregation into tasks. This adaptation permits the categorization of the older population into severity groups of dependency established by the SNIC. The BAREMO adaptation has been validated in a report that found that the adaptation identifies accurately the severe-and moderate-dependent but works worse for the mild- and non-dependent population [22]. Furthermore, the dependency severity groups obtained with the BAREMO may not fully capture the heterogeneity of dependency. The new World Health Organization (WHO) classification of functioning includes limitations in different types of activities, such as self-care, mobility, or domestic activities (e.g. preparing food) [16, 23], and dependency may be related to any of these activities. Hence, gaining a better understanding of the diversity of activities in which assistance is required may contribute to improving the knowledge about the heterogeneity of dependency. Therefore, improved profiles of dependency may be useful input in the design of services that adequately cater for the needs of individuals with different needs. In our study, we deepen the understanding of the population of older adults in Uruguay who are dependent, emphasizing the differences according to the type of activities in which they require help and improving the knowledge about the type of assistance they required.

Our aim is to identify groups of dependent older adults living in Uruguay and their relationship with sociodemographic characteristics using a latent class approach. We consider the state of dependency as a latent state, and identify groups based on the declared need for help to perform ADL. We also characterized the groups according to the types of activities in which people require help (BADL, IADL and/or AADL), and by sociodemographic characteristics (age, sex, and education). Last, we compare these latent dependency classes with the classification obtained by the adapted BAREMO scale, which is used by Uruguay’s national care program to allocate long-term care benefits.

Previous publications have used the latent construct approach to analyse the health status and disability of the older population [24–27]. Yet, they focus on people living in developed countries. There is limited literature on dependency understood as the need for help to perform ADL. For instance, a previous analysis [25] focus on the relation between self-perceptions of ageing and latent health trajectories, assessed by the difficulties to perform ADL, with data from the Health and Retirement Survey (HRS) from United States. Different patterns of aging-related health trajectories were reported and this analysis highlighted the multidimensionality of the health construct and the heterogeneity of ageing. Further, it emphasized that the deterioration of health with ageing does not follow a single pattern. Other work [24] also applied a latent variable approach to the health of the older population analysing the transitions among states of difficulties in performing ADL over time and reported the heterogeneity in health with aging. Another analysis [26] identified disability profiles of older Italian adults using a similar analytical approach. With a cross-sectional approach, they used information about the functional limitations and difficulties in performing ADL and reported heterogeneity of disability in older adults. For people over 65 years in England, a previous analysis [27] applied structural equations modelling to model disability as a latent variable and studied how it influenced the allocation of a disability transfer. The authors used three representative surveys with different sets of covariates to assess disability, including a combination of ADL and functional limitations. They report that the use of a latent variable approach allows them to take advantage of the existence of multiple indicators, while avoiding the problems of constructing ad hoc indices or over-interpreting potentially unreliable indicators.

Finally, the literature that uses a latent approach of health, disability or dependency in non-developed countries is scarce although there is a recent work applied to the older Mexican population [28]. In this study, the authors used cross-sectional data and applied a multidimensional and broad definition of dependency. The authors reported that only variables associated with physical limitations played a relevant role in the classification among dependency groups.

This brief review of the literature that analysed health conditions, disability and dependency in the older population illustrates the complex interrelated network of concepts and dimensions. Previous literature reported that there is heterogeneity in disability, dependence or health status captured in more than two latent classes [24–27], but these results cannot be generalized to different contexts or data sources as the methodology is data-driven and results are dependent on analytical decisions made in each study as well as sample and study design characteristics.

The contribution of this work, is to use a latent approach to analyse dependency in older population, carefully conceptualizing this construct as the need for help to perform ADL, as considered by Harwood, Sayer and Hirschfeld (2004) [13] and care programs, particularly in Uruguay. In the latent approach, we consider the need for help to perform different types of ADL as the observable variables. As mentioned before, previous literature does not focus on dependency as the need for human help, yet much of the literature is interested on disability or health status. Additionally, our approach allows us to relate the dependency profiles, results of the latent approach, with the classification used by the SNIC of Uruguay, that provides benefits to dependent older population and that may be one of the models to be followed by other Latin American countries. Moreover, to identify if there is heterogeneity of the dependency of older population that may be related to the type of care needed, is important to understand the met or unmet care needs of this population, that may have adverse consequences on the quality of life of this people and their families [29, 30].

Further, most of the previous studies have considered health or disability as latent variables in the context of developed countries, thus we apply similar methodologies in a non-developed country.

## Methods

### Data

We used data from the *Encuesta Longitudinal de Protección Social* (ELPS) for Uruguay. Baseline data were collected between 2012 and 2013, and the follow-up assessments between 2015 and 2016. The sampling frame for baseline data was the 2004 Population Census of Uruguay and a stratified sampling method was used to obtain a representative sample of the national population of individuals aged 14 years old and older living in particular homes. The interviews were conducted at home and the information is self-reported [31]. Participants were asked about their sociodemographic characteristics and health condition, in addition about whether they received government benefits, their employment history, social security, heritage and household composition. Specifically, participants were asked their age, sex and the highest level of education. Participants aged 60 years or older were asked whether they had difficulties to perform 11 ADL and if they do so if they need help to perform these activities.

We considered individuals aged 60 years old and older at the follow-up survey conducted in 2015/16 when the SNIC policies began to be implemented. Of the 6197 individuals in this age group surveyed at baseline, 1716 (27.7%) were not included in the follow up wave (10.4% died, 1.3% moved to a nursing home and 16.1% were untraceable). Also, 657 (13% of the final sample) were included because they reached 60 years old in the period between baseline and follow-up. Hence, the analytical sample included 5138 individuals with complete data in the follow-up survey. For further information about the design and the data collection of the ELPS see [31].

### Variables and measures

For the purposes of our analyses, we derived a series of dummy variables to account for differences in education (1 = the educational attainment is higher than primary school, secondary or tertiary), 0 = the educational attainment is primary school or less) and sex (1 = Female, 0 = Male). Age was measured in years.

To assess dependency we derived 11 dummy variables that indicate whether the person needs help to perform a daily activity. We considered the activities asked in the ELPS, which were also included in the adaptation of the BAREMO scale, and include basic, instrumental, and advanced ADL, specifically: eating, dressing, personal care activities (tooth brushing, combing or face washing), displacing inside home (e.g. walking around a room), using the restroom, avoiding health risk (e.g. taking medications), changing/maintaining position, displacing outside home (e.g. walking outside the home), performing housework, participating in social life and communicating. Each dummy variable takes the value 1 if the individual needed help with the activity on a regular basis (i.e. declared the need for help ‘Sometimes’, ‘Many times’ or ‘Always’), and 0 otherwise.

### Statistical approach

We fitted latent class regression models [32] to analyse dependency of the older adults. In this approach, the outcome (dependency) is a latent categorical variable which is expressed visibly by other manifest variables. In our case, the manifest variables were the 11 dummies which indicate the need for help in the different ADL.

In this framework, it is assumed conditional independence of the outcomes given the class membership to compute the probability that an individual *i* in the class *r* produces a particular set of *j* outcomes on the manifest variables. An specific outcome *Y*_*ij*_ equals one if respondent *i* needs help to perform the *j*_*th*_ activity and zero otherwise, and *π*_*jr*_ denotes the class-conditional probability that an individual in the class *r* needs help to perform the *j*_*th*_ activity. To characterize the latent dependency classes by sociodemographic characteristics, the latent class regression were adjusted by covariates (*X*_*i*_), particularly, by age, sex, and educational attainment, including the interaction between age and sex.

The number of categories of the latent outcome, i.e. the number of classes, is unknown. Thus, we fitted the models for one to five classes and selected the number of classes that best fit the data, using the Bayesian Information Criterion (BIC), the consistent Akaike Information Criterion (cAIC), and the Akaike Information Criterion (AIC). Additionally, the entropy index is calculated in order to assess how good is the separation among the latent classes identified and the Lo-Mendell-Rubin likelihood test is performed to provide further evidence on the number of latent classes that best fits the data. We also considered the conceptual interpretation of the dependency classes to select the final number of classes [32, 33].

Once the classes have been identified, we computed the conditional probability that an individual that belongs to a specific latent class *r*, produces a given value in each of the manifest variables *j* (${\hat{\pi}}_{jr}$). In our context, these estimates are the expected probabilities of needing help for each activity, conditional on class membership. Based on these results we interpret the need-for-help profiles associated with each latent dependency class, considering the type of activities in which it is more likely to require help conditional on belonging to a given class.

In addition, we estimated the posterior probabilities that an individual belongs to each class conditional on their observed values of the manifest variables and covariates in order to characterize the identified latent dependency classes in terms of the sociodemographic characteristic.

We used the poLCA library from R to estimate the latent class regressions (Linzer and Lewis 2011) [34].

### Analysis of the relation between the latent classes and the BAREMO classification

As mentioned, the BAREMO instrument was adapted to measure dependency in the population-based study of Uruguay ELPS. We classified the individuals according to this adaptation of BAREMO scale and compare with our latent dependency class assessment.

The adaptation of the BAREMO instrument considers 11 activities, out of the 13 included in the original measure, that were asked in the ELPS survey. For each individual to whom the adapted instrument is applied, it determines the need for help in the ADL, the type of help needed (verbal indication, complete substitution of the activity) and the frequency of help needed (always, sometimes, rarely, etc.) [21, 22]. Briefly, the BAREMO adaptation assigns a score to the 11 ADL according to the need for help to perform the activity, and as a result a value on a continuous scale is obtained (range 0–100). The SNIC of Uruguay have determined cut-off points for classifying into degrees of severity of dependence: non-dependent, mildly dependent, moderately dependent, and severely dependent [21, 22]. The cut-off points are indicated in Fig. S1 of the Supplementary material.

After we classify the individuals on each latent class, we apply the BAREMO adaptation and classify the individuals of our analytical sample into the severity dependency groups defined by this scale. Then, we analyse the value of this scale across individuals of the different latent dependency classes comparing with the classification with the BAREMO adaptation and the latent dependency classes.

## Results

### Descriptive statistics

Descriptive characteristics of the sample are presented in Table 1. The average age of individuals in the sample was 72.4 (SD = 8.5) years old and 62% were women. 45% of the individuals reported a higher educational attainment (completed more than primary school level).

**Table 1:** Descriptive statistics of analytical sample (*N* = 5138)

*Age*	Mean (SD)	*Sex*	N (%)
Age (years)	72.4 (8.5)	Female	3173 (61.8)
*Age ranges*	N (%)	Male	1965 (38.2)
60–64	1096 (21.3)		
65–69	1081 (21.0)	*Education*	N (%)
70–74	997 (19.4)	Primary	2799 (54.5)
75–79	832 (16.2)	Secondary	1645 (32.0)
80–84	635 (12.4)	Tertiary	694 (13.5)
85+	497 (9.7)		
*Need for help in ADL*	N (%)		
Eating	54 (1.1)	Avoiding health risk	158 (3.1)
Dressing	197 (3.8)	Displacing outside home	535 (10.4)
Displacing inside home	179 (3.5)	Performing housework	501 (9.8)
Changing/maintaining position	180 (3.5)	Participating in social life	227 (4.4)
Using the restroom	167 (3.3)	Communicating	137 (2.7)
Personal care activities	217 (4.2)		

Descriptive statistics of the main variables and measures in the analytical sample

The analytical sample (*N* = 5138) did not differ from individuals at baseline with 60 years old or older in terms of age (t-test, *p* = 0.054) and sex (t-test, *p* = 0.292). However, there are differences in terms of education (t-test, *p* = 0.000), as individuals in the analytical sample have a higher educational attainment than individuals in the baseline sample.

### Identification of the dependency classes

We fitted latent class regressions including age, sex, and education by setting the number of classes from one to five. According to the values of the BIC, the cAIC, and the AIC four latent classes were identified (see Table S1 in supplementary material). Furthermore, the separation into four classes also seems to be conceptually appropriate since the distribution of individuals across classes keeps a reasonable minimum of cases for all latent classes. The Entropy index for the four-class model was 0.916, indicating a good separation of the population into these groups, given that the practical recommendation is for this index to be 0.8 or higher [33] and the Lo-Mendell-Rubin likelihood statistic supports the separation in four latent classes. Additionally, the average latent class posterior probability for the four latent classes, where all diagonal values are greater than 0.848 and off-diagonal elements are closer to zero, as is desirable [33] (see Table S1 in supplementary material).

### Profiles of the need for help with the ADL for the dependency classes

**Fig. 1:**
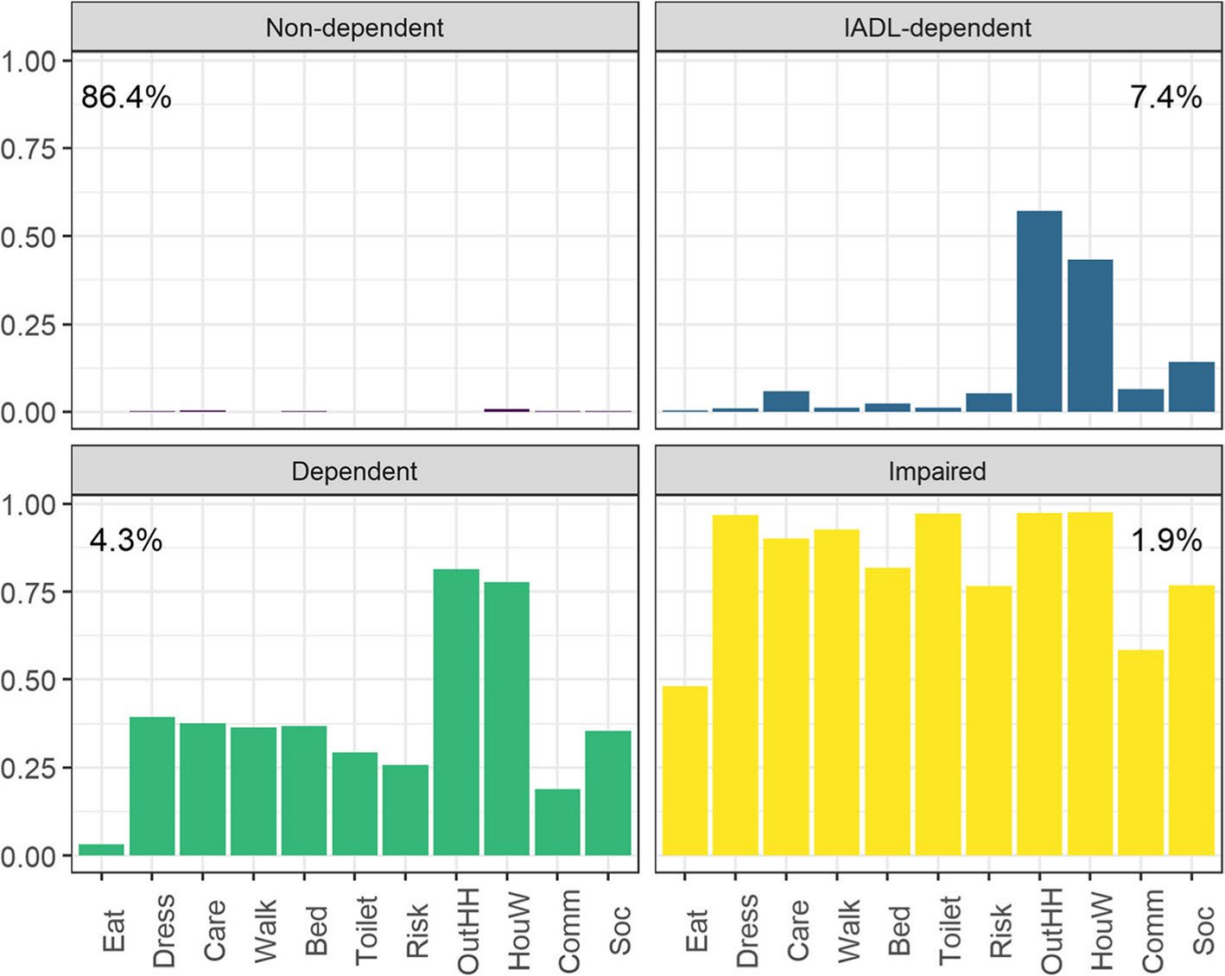
Expected probability of needing help in each activity conditional on class membership. Notes: IADL: Instrumental Activities of Daily Living; Eat: eating; Dress: dressing; Care: personal care activities; Walk: displacing inside home; Bed: changing/maintaining position; Toilet: using the restroom; Risk: avoiding health risk; OutHH: displacing outside home; HouW: performing housework; Comm: communicating; Soc: participating in social life

The results showed large heterogeneity among these four classes regarding the type of activities in which its members require help. Figure 1 depicts the expected probabilities of needing help for each of the 11 activities, conditional on the class membership. Over 86.4% of the individuals were classified in the class we call ‘Non-Dependent’ (ND), in which people have a very low expected probability of needing help for any of the ADL (see Fig. 1).

In turn, the remaining 13.6% were classified into the other three classes, and people assigned to these classes are expected to need help to perform at least one ADL, although different types of activities and in different degrees. As shown in Fig. 1, the ‘IADL-dependent’ class represents 7.4% of the sample, and individuals assigned to that class have a high expected probability of needing help in two instrumental activities: displacing outside home and performing housework. The ‘Dependent’ class constitutes 4.3% of the individuals, and they are expected to require assistance with basic, instrumental, and advanced activities, except for eating. Finally, individuals assigned to the ‘Impaired’ group are expected to need help in all activities and represent 1.9% of the sample. Their expected probability of needing help with eating in this group is almost 0.5.

### Sociodemographic characteristics of the dependency classes

To analyse differences in sociodemographic characteristics across the dependency classes, we included various covariates in the latent class regressions (age, sex, the interaction between age and sex, and an indicator of high educational attainment).

Older age is significantly associated with assignment to classes that have some type of dependency in ADL, i.e., ‘IADL-dependent’ (*p* < 0.001), ‘Dependent’ (*p* < 0.001) and ‘Impaired’ (*p* = 0.003) relative to the ‘Non-dependent’ class (see Table S3 in supplementary material). In addition, being a woman is a significant factor for being classified into the classes with some type of dependency relative to the ‘Non-dependent’. Additionally, women have a different effect of age in the probability of being assigned into the ‘IADL-dependent class (*p*=0.038) and into the ‘Impaired’ class (*p*=0.005), though the interaction between age and sex is not statistically significant for the probability of being in the ‘Dependent’ class with respect to the ‘Non-dependent’ class (*p* = 0.603). Finally, a higher level of education reduces the probability of classification into the dependency groups (*p* = 0.035 for ‘IADL-dependent’ and *p* = 0.031 for ‘Impaired’), although statistical significance was not reached for the ‘Dependent’ group with respect to the ‘Non-dependent’ group (*p* = 0.200).

**Fig. 2:**
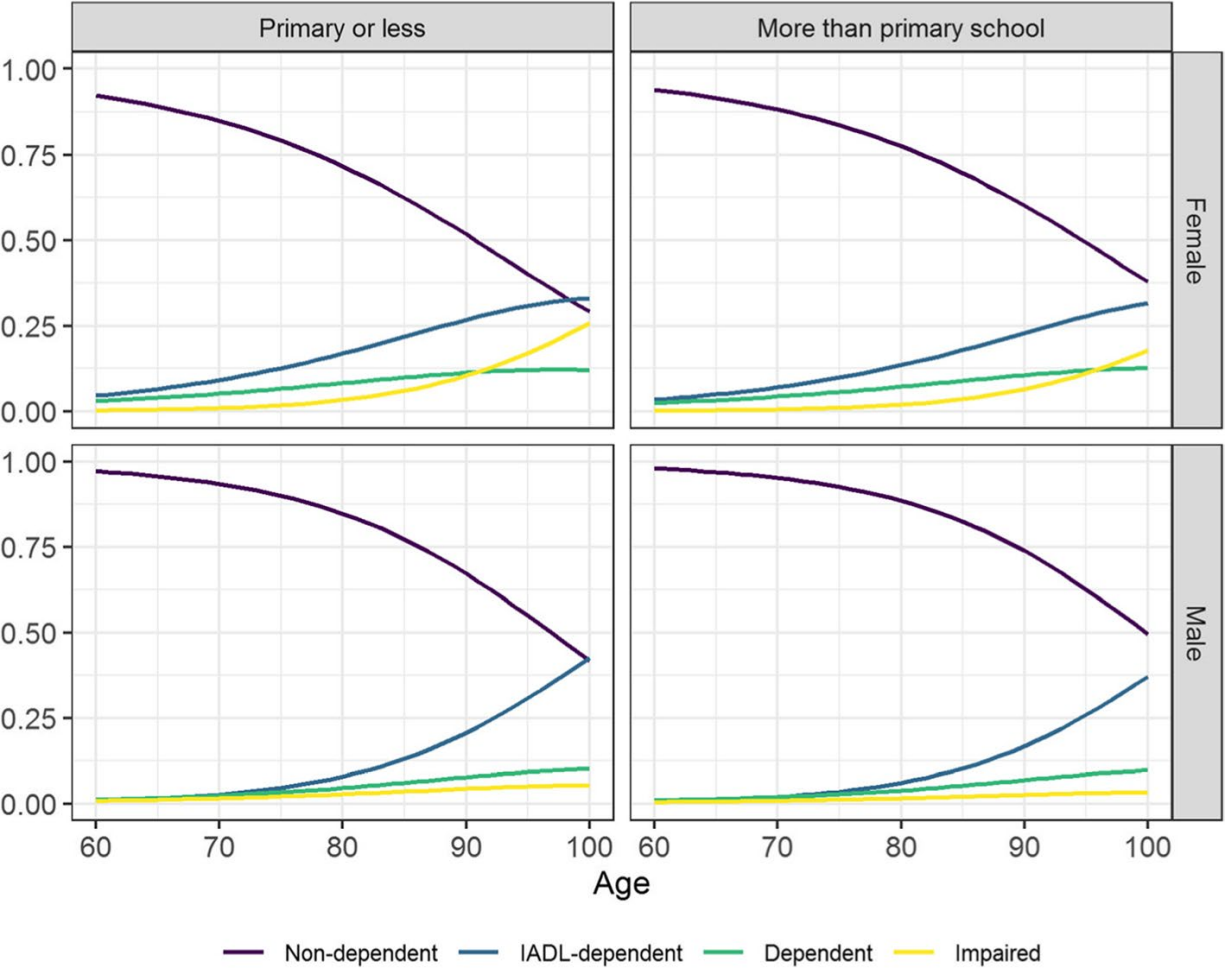
Probability of being assigned to each class according to age, sex, and education level. Notes: The latent dependency classes identified are: ‘Non-dependent’, ‘IADL-dependent’, ‘Dependent’ and ‘Impaired’

We synthesize how the estimated probabilities of being assigned to each latent class vary with sociodemographic characteristics in Fig. 2, showing these probabilities according to age, sex and educational attainment. The probability of being assigned to the ‘Non-dependent’ class decreases with age, regardless of sex and educational levels. Notably, there is a great decrease on this probability for people aged 80 years or older.

Nonetheless, the results showed sex differences in the probability of being classified into each of the three dependency classes. For instance, the probabilities of being assigned to the ‘IADL-dependent’, ‘Dependent’, or ‘Impaired’ classes are almost zero for men under their 80 years old, for both educational levels. However, these probabilities are higher for women of at least 80 years old and are even higher for those with lower educational attainment. In particular, the results showed a higher probability of being classified into the ‘IADL-dependent’ class for younger women with a lower educational attainment (see Fig. 2).

### Association of the four latent dependency classes with the BAREMO scale

We analysed the BAREMO scale values of the individuals according to their classification into the four latent dependency classes. The BAREMO adaptation is a continuous scale with cut-off points used to classify individuals into four dependency severity groups: non-dependent, mild-dependent, moderate-dependent and severe-dependent.

**Fig. 3:**
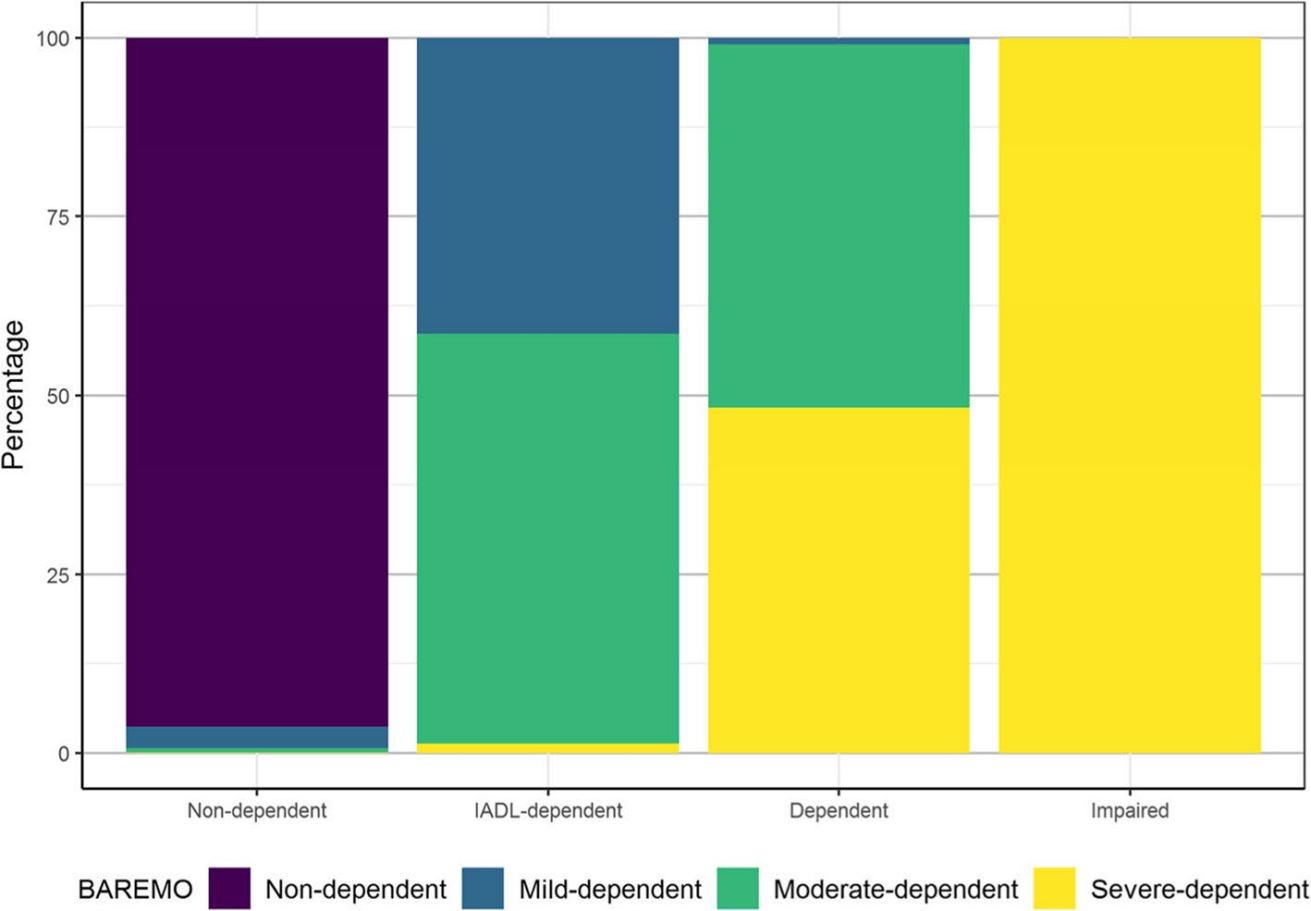
Overlap of classification in four latent classes and the adaptation of BAREMO scale. Notes: Latent classes identified are: ‘Non-dependent’, ‘IADL-dependent’, ‘Dependent’ and ‘Impaired’. The degrees of dependency with the BAREMO are depicted by colours: ‘Non-dependent’, ‘Mild-dependent’, ‘Moderate-dependent’ and ‘Severe-dependent’

Figure 3 depicts the comparison between the classification of BAREMO adaptation and the latent dependency classes identified, and the results showed that there is almost a perfect match between the ‘Non-dependent’ class and the individuals classified as non-dependent with the BAREMO adaptation scale. However, although all individuals in the ‘Impaired’ class are severely dependent according to BAREMO, there are also some severely dependent individuals classified into other latent classes of dependency. Almost 51% of the ‘Dependent’ class are moderately dependent, 48% are severely dependent, and only 1% are mildly dependent according to the BAREMO adaptation. Finally, 1% of the ‘IADL-dependent’ class are severely dependent, 57% are moderately dependent, and 41% are mildly dependent according to BAREMO.

Finally, the overlap of the four latent dependency classes with the BAREMO score suggests an ordering in terms of severity of dependence of the identified latent classes according to the BAREMO measure (see Fig. S1 in the supplementary material).

### Sensitivity analysis

We performed sensitivity analyses to study the robustness of our results. To check the separation of the sample into the four identified latent classes, we first considered different dummies to indicate educational attainment (Supplementary material, Table S3 and Table S4) and we also fit the latent class regressions without including covariates (Supplementary material, Table S5). The results showed the robustness of the heterogeneity in the profiles of dependency of the older adults in terms of the types of ADL in which the individuals require help. Moreover, the results are robust to the identification of four latent dependency classes, however there was an exception in which the models identified three latent dependency classes. We analysed the reclassification of individuals across classes between the model of three and four classes, and changes were minimal.

## Discussion

The aim of this study was to analyse the dependency situation of the community-dwelling older population from Uruguay using a latent approach. We identified the existence of heterogeneous groups of dependent older population and analysed their profiles in terms of the need for help in different types of ADL. The latent class regression models were adjusted to synthesize the responses on the need for help in 11 ADL asked in the nationally representative study ELPS from Uruguay. In our analyses, 13.6% of the individuals were identified as dependent but large differences emerged in the type of activities in which they needed help. Also, we assessed the role of age, sex and education on the probability of belonging to the different dependency classes, and the results showed heterogeneity in these probabilities according to sociodemographic characteristics. Finally, we relate the classification resulting from the latent class analysis to the one derived from the adaptation of the official scale to the nationally representative ELPS study. This sheds light on the different types of activities in which help is most likely to be required in relation to the degree of severity of the BAREMO adaptation.

In keeping with previous studies, our results supports that older population have different patterns of aging, resulting in the heterogeneity of health conditions, disability and dependency [24–28]. The strategy of considering dependency as a latent variable allowed us to separate dependent people into differentiated groups in terms of the need for help and, therefore, the type of care they should receive to meet their needs. Accordingly, our work shows that among people who need help there is diversity in the type of activities in which they require help. Thus, half of the individuals with help requirements mainly need it to perform instrumental activities while the other half need help to perform all type of activities. Moreover, within this subgroup of individuals with needs in all type of activities, there exists a smaller group of impaired older adults with high probability of needing help to eat (1.9% of the sample).

These results highlight the existence of heterogeneity in dependency of people living at home. The different needs should lead to the provision of different types of service or assistance that meet the specific needs that these individuals may have. It is therefore important for caregivers and decision makers to take these heterogeneities into account to avoid the negative effects of not meeting the needs of dependent older people [29, 30].

Our results are in line with previous work that posits a hierarchy in the loss of functionality as people age. In particular, it was suggested that the loss of capacity during aging occurs in the reverse order in which these activities are learned in childhood [35, 36]. Thus, people in the ‘Impaired’ class are most likely to need help with eating and are the most aged group of people, with a significant share of people over their 80 years old in that group.

Additionally, our classification of individuals into the different latent dependency classes has similarities with previous work conducted in developed countries. In those studies, the health status or disability of the older adults were reported as heterogeneous, and our results showed a similar pattern of the proportion of individuals in the different classes [24, 26]. This pattern suggests a high percentage of older people living at home with a good health condition, without disability or dependency. But, at the same time, there is a non-negligible percentage of older people living at home (between 2 and 7% depending on the study) with important functional limitations, which may result in the need for daily help from others.

Further, our results showed significant differences among dependency classes according to sociodemographic characteristics. This highlights the sociodemographic differences related to the dependency profiles in terms of the types of activities in which people require help, hence the differences in the assistance needed by the older population. In our analyses, emerged a group of people which mainly need help in instrumental activities (‘IADL-dependent’), and belonging to that group is more likely for women with low educational attainment. These results show that there is a type of dependency that may not be addressed by the current services provided by the SNIC of Uruguay, and it would be desirable to consider this type of dependency to improve the quality of life of these individuals and their families. Besides, this may respond to a gender division of tasks inside the household, where men do not perform this type of activities and therefore do not report having difficulties or needing help to perform them in later life, which is in agreement with the results reported in previous literature [37]. Additionally, being a woman is a significant factor for belonging to the classes with some type of dependency relative to the ‘Non-dependent’. In particular, the results showed a higher probability of being in the ‘IADL-dependent’ class for younger women with at most primary school education. This evidence is in agreement with the “male-female survival health paradox” since women live longer and are more likely to need for help in all ADL than men [38].

To inform care policies in Uruguay, we finally relate our classification to the degrees of severity obtained by applying the BAREMO adaptation, which constitutes the official scale used by the SNIC to assign benefits. First, our results showed an association between the severity groups of dependency from BAREMO and the type of activities in which is more likely to need help. For instance, individuals in the ‘Impaired’ group of our latent class approach are mostly severely dependent for the BAREMO adaptation while the ‘Dependent’ individuals are mostly moderate and severe dependents. In particular, individuals in the ‘IADL-dependent’ class, whose needs are more likely to be in instrumental activities, are mostly mild and moderate dependents. Thus, the help provided to individuals with these degrees of dependency, should consider that these individuals are more likely to need help with displacing outside home and performing housework. Therefore, it would be useful to diversify the type of care programs provided to the dependent population. For example, currently daily help is provided to severe dependents, which are individuals with needs in all types of activities, but perhaps it would be desirable to have some service that provides help specifically for these two instrumental activities (displacing outside home and performing housework) that are the most predominant for all severity degrees of dependency. In addition, the services currently provided for individuals with mild or moderate dependence may be underused if people have no way of accessing them, for example, because they cannot go to Day Care Centres because they need help that is not available to them.

Our results are not exempt from limitations. The cross-sectional analysis hampered the consideration of the dynamics of dependency. Although the ELPS has two waves of gathered information, previous work found difficulties in applying dependency measures such as the BAREMO adaptation, consistently between both waves. Also, the latent approach of dependency using only two points in time, would be difficult to implement. The methodological approach with a longitudinal perspective needs sufficient variability in the transitions that is difficult to obtain with maximum two occasions per individual. Using the follow-up survey of the ELPS study adds a potential selection bias, given the loss of individuals in our analytical sample, particularly due to death. Our results showed that individuals in our analytical sample have higher educational attainment than individuals in the baseline survey. This could be affecting the relationship between latent dependency classes and educational attainment. Future work particularly interested in this relationship should consider methods that address this potential problem. However, despite these limitations our analysis gives new insights about the ageing in a developing country with an older population structure, that has implemented a framework in which health and social care policies are displayed.

## Conclusions

Our results suggested the different dependency profiles in terms of the type of activities in which help is needed. This is an important contribution that informs to the design of care policies and the diversification of the assistance provided to community-dwelling older population, in the context of developing countries with resources constraints. Future work should explore the typology of latent classes and the severity of dependency to anticipate the care expectations of dependents and to be able to adjust the supply of public policies accordingly. People with the same degree of dependency may need or want different types of care. While severity should order the priority of different interventions, diversifying supply based on the type of assistance needed rather than severity only, may be a good strategy to consider.

## Supplementary Information

**Additional file 1.**

## Data Availability

The data that support the findings of this study are available from *Banco de Previsión Social* but restrictions apply to the availability of these data, which were used under license for the current study, and so are not publicly available. Data are however available from *Banco de Previsión Social* upon reasonable request (send request to *Banco de Previsión Social*, email: pedidoelps@gmail.com). For further information to request the data from this study contact AM (email: alejandra.marroig@fcea.edu.uy).

## Abbreviations

ADL: Activities of Daily Living
BADL: Basic Activities of Daily Living
IADL: Instrumental Activities of Daily Living
AADL: Advanced Activities of Daily Living
SNIC: *Sistema Nacional Integrado de Cuidados* (The National Integrated Care System)
BAREMO: *Baremo de dependencia*
ELPS: *Encuesta Longitudinal de Protección Social* (Longitudinal Social Protection Survey)
