# Supplementary material for: Latent dependency classes according to the need for help: a population-based analysis for the older population
Source: BMC Geriatr. 2022 Jul 26;22:621. doi: 10.1186/s12877-022-03307-w (PMC9317231; doi:10.1186/s12877-022-03307-w)
Supplement: Supplementary file 1 — Additional file 1. [file 12877_2022_3307_MOESM1_ESM.docx]

## Supplementary material

Table S1 Goodness of fit and mean posterior probability for four latent classes

| # classes | log-likelihood | BIC | cAIC | AIC | Entropy | LMR |  |
| --- | --- | --- | --- | --- | --- | --- | --- |
| 1 | -9953.45 | 20000.89 | 20011.89 | 19928.90 |  |  |  |
| 2 | -6154.01 | 12538.71 | 12565.71 | 12362.01 | 0.970 | 7313.56^(*)^ |  |
| 3 | -5855.59 | 12078.59 | 12121.59 | 11797.18 | 0.953 | 574.42^(*)^ |  |
| 4 | -5605.96 | **11716.05** | **11775.05** | **11329.93** | 0.916 | 480.51^(*)^ |  |
| 5 | -5854.93 | 12350.70 | 12425.70 | 11859.86 | 0.970 | -479.24 |  |
| Mean posterior probability conditional on class membership for four latent classes | | | | | | | |
| Class | P(C=1 \| C=j) | P(C=2 \| C=j) | P(C=3 \| C=j) | P(C=4 \| C=j) | N | % | |
| 1 | 0.979 | 0.021 | <0.001 | <0.001 | 4441 | 86.43 | |
| 2 | 0.059 | 0.848 | 0.093 | <0.001 | 380 | 7.40 | |
| 3 | 0.001 | 0.093 | 0.883 | 0.022 | 221 | 4.30 | |
| 4 | <0.001 | <0.001 | 0.066 | 0.934 | 96 | 1.87 | |

Notes: # classes: Number of classes; P(Class=k | Class=j): is the mean probability of belonging to k-th class given that the individuals are classified in the j-th class; LMR: Lo–Mendell–Rubin statistic and ^(*)^ indicates p-value < 0.05.

Table S2 Associations of sociodemographic characteristics among the dependency classes

|  | ‘IADL-dependent’ vs. ‘ND’ | | ‘Dependent’ vs. ‘ND’ | | ‘Impaired’ vs. ‘ND’ | |
| --- | --- | --- | --- | --- | --- | --- |
|  | Sign | p | Sign | p | Sign | p |
| Age | + | <0.001 | + | <0.001 | + | 0.003 |
| Sex | + | 0.008 | + | 0.333 | - | 0.007 |
| Age*Sex | - | 0.038 | - | 0.603 | + | 0.005 |
| >Primary | - | 0.035 | - | 0.200 | - | 0.031 |

Notes: sign and corresponding p-values of the effects of covariates on the probability of belonging to the latent classes ‘IADL-dependent’, ‘Dependent’ and ‘Impaired’ with respect to the probability of belonging to the ‘Non-dependent’ latent class.

Figure S1 Density of the BAREMO scale and its cut-off points for severity of dependency according to the classification into the four latent dependency classes.


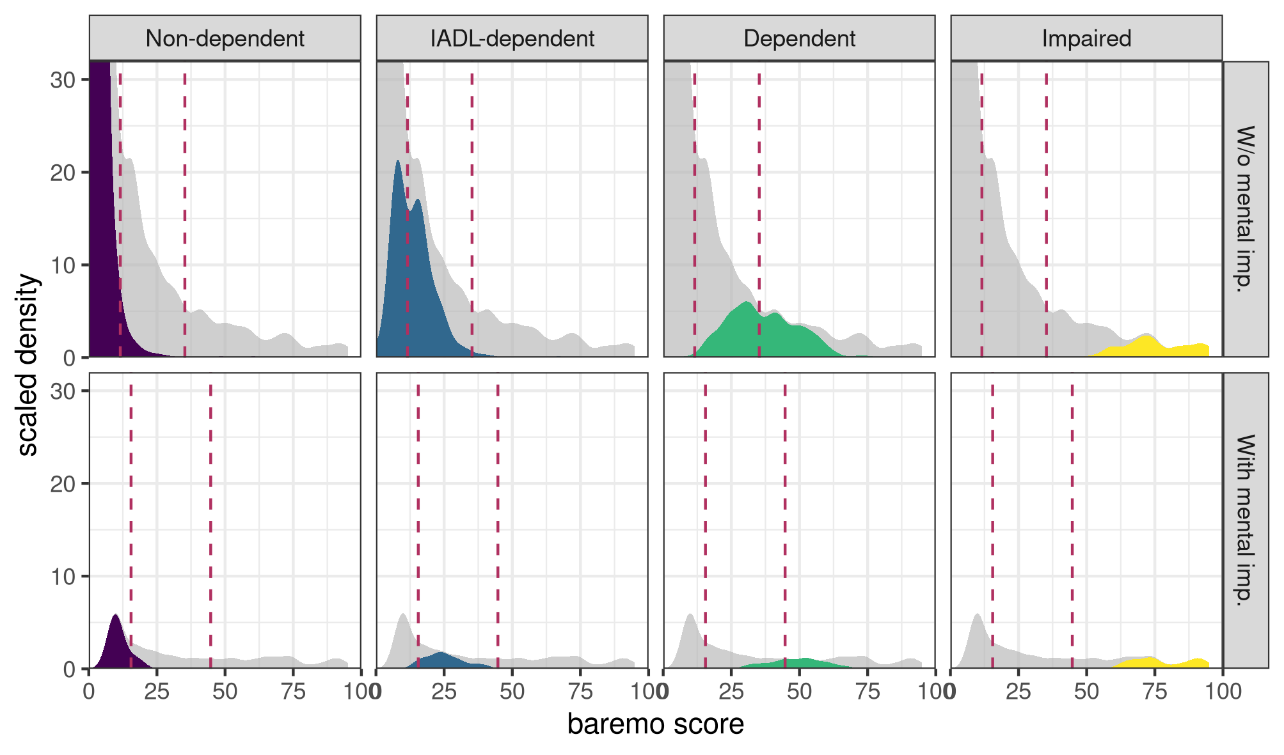


Notes: Only includes individuals with positive score in the BAREMO evaluation (~17 per cent of the sample). Red lines indicate the cut-off points between categories. A person with a score of zero on the scale is classified as non-dependent, with a positive score below 11.56 as mildly dependent, with a score between 11.56 and 35.24 as moderately dependent, and with a score above 35.24 as severely dependent. If the person declares to have a mental condition, the cut-off points differ slightly such that zero is still classified as non-dependent, a positive score below 15.51 as slightly dependent, a score between 15.51 and 44.72 as moderately dependent and a score above 44.72 as severely dependent (21).

Table S3 Goodness of fit of education in three categories: primary/secondary/tertiary

| N. classes | log-likelihood | BIC | cAIC | AIC | Entropy | LMR |
| --- | --- | --- | --- | --- | --- | --- |
| 1 | -9953.45 | 20000.89 | 20011.89 | 19928.90 |  |  |
| 2 | -6151.61 | 12542.47 | 12570.47 | 12359.23 | 0.970 | 7318.2^(*)^ |
| 3 | -5688.46 | 11761.41 | 11806.41 | 11466.91 | 0.954 | 891.53^(*)^ |
| 4 | -5601.17 | **11732.10** | **11794.10** | 11326.35 | 0.913 | 168.01^(*)^ |
| 5 | -5551.76 | 11778.54 | 11857.54 | **11261.53** | 0.921 | 95.11^(*)^ |

Notes: LMR: Lo–Mendell–Rubin statistic and ^(*)^ indicates p-value < 0.05.

Table S4 Goodness of fit of tertiary education dummy

| N. classes | log-likelihood | BIC | cAIC | AIC | Entropy | LMR |
| --- | --- | --- | --- | --- | --- | --- |
| 1 | -9953.45 | 20000.89 | 20011.89 | 19928.90 |  |  |
| 2 | -6151.91 | 12534.51 | 12561.51 | 12357.81 | 0.970 | 7317.6^(*)^ |
| 3 | -5691.56 | 11750.53 | 11793.53 | 11469.12 | 0.954 | 886.12^(*)^ |
| 4 | -5605.00 | **11714.13** | **11773.13** | 11328.00 | 0.915 | 166.61^(*)^ |
| 5 | -5555.59 | 11752.01 | 11827.01 | **11261.18** | 0.923 | 95.12^(*)^ |

Notes: LMR: Lo–Mendell–Rubin statistic and ^(*)^ indicates p-value < 0.05.

Table S5 Goodness of fit of model without covariates

| N. classes | log-likelihood | BIC | cAIC | AIC | Entropy | LMR |
| --- | --- | --- | --- | --- | --- | --- |
| 1 | -9953.45 | 20000.89 | 20011.89 | 19928.9 |  |  |
| 2 | -6284.85 | 12766.22 | 12789.22 | 12615.7 | 0.968 | 7061.7^(*)^ |
| 3 | -5861.21 | 12021.47 | **12056.47** | 11792.41 | 0.963 | 815.47^(*)^ |
| 4 | -5808.56 | **12018.72** | 12065.72 | 11711.13 | 0.944 | 101.33^(*)^ |
| 5 | -5767.15 | 12038.42 | 12097.42 | **11652.3** | 0.934 | 79.71^(*)^ |

Notes: LMR: Lo–Mendell–Rubin statistic and ^(*)^ indicates p-value < 0.05.
